# Hybrid surgery management challenges of a Behcet's disease patient with recurrence of aortic aneurysms: a case report

**DOI:** 10.3389/fcvm.2023.1097129

**Published:** 2023-09-01

**Authors:** Guo Xin Chen, Jiang Hong Wan, Chu Wen Chen, Bin Huang

**Affiliations:** ^1^ Department of Vascular Surgery, West China Hospital, Sichuan University, Chengdu, China; ^2^ Department of Outpatient, West China Hospital, Sichuan University, Chengdu, China

**Keywords:** behcet’s disease, hybrid surgery, recurrent aneurysms, endovascular aneurysm repair, open surgery repair, revascularization, bypass

## Abstract

**Background:**

Behcet's disease is a vasculitis of unknown origin that can involve multiple organs or tissues. Aneurysm or pseudoaneurysm, also one of the complications of Behcet's disease, is usually accompanied by a poor prognosis. Surgery is usually accompanied by a high risk of complications, such as the recurrence of anastomotic pseudoaneurysms and blockage of the target vessel. Using hybrid surgery, we successfully treated a complex and recurrent abdominal aortic pseudoaneurysm in a patient with BD.

**Methods:**

We report a 32-year-old female diagnosed with Behcet's disease with recurrent thoracoabdominal aortic aneurysm. Adequate immunotherapy was given during the perioperative period. The splanchnic artery branches were reconstructed, and the aneurysm was sequestered with endovascular repair. The patient recovered uneventfully and was discharged from the hospital 8 days after hybrid surgery. At the 60-month follow-up, no aneurysm was observed, the stent had no displacement or internal leakage, and the reconstructed blood vessels were unobstructed.

**Conclusion:**

Hybrid surgery could be a feasible and effective strategy for BD aneurysms. Adequate preoperative and postoperative immunotherapy with arterial anastomosis away from the diseased artery may be the key to success.

## Introduction

Aortic aneurysms are common in elderly individuals but rare in young individuals and are mostly caused by vascular tissue degradation, trauma, inflammation, infection, fibromuscular dysplasia and Marfan syndrome. Recurrent aneurysms are relatively rare, especially in young people. Recurrent aneurysms have been reported in Behcet's disease (BD) (1, 2), which is a systemic variable vessel vasculitis that involves the skin, mucosa, joints, eyes, arteries, veins, nervous system and the gastrointestinal system. BS mainly occurs in countries located between 30 and 45° north latitude through the Mediterranean basin, the Middle East, and the Far East regions, but it is rare in Europe and North America (3). It is the only primary vasculitis that affects both arteries and veins of any size (4). However, arterial lesions are relatively rare and manifest mostly in the form of aneurysms (5). BS is commonly seen in patients aged 20 and 40 years and has a male preponderance with a male to female ratio of 5.02 in patients with vascular involvement, although the overall patient ratio is 4.4 (6, 7). Medical treatment with cyclophosphamide and corticosteroids is necessary before intervention to repair both aortic and peripheral artery aneurysms. Surgery or stenting should not be delayed if the patient is symptomatic, according to the 2018 ELUAR recommendations (8).

Treatment for aortic aneurysms involving the visceral arteries is a difficult surgical challenge. Surgery is still the prime treatment for juxtarenal aortic aneurysms (9–11), while in today's minimally invasive era, approximately 80% of patients undergo endovascular aneurysm repair (EVAR) or fenestrated or branched EVAR surgery (12–14). Our case aimed to report hybrid surgery for a patient with recurrent abdominal aneurysm and Behcet's disease and to conduct a literature review.

## Case presentation

**Figure 1:**
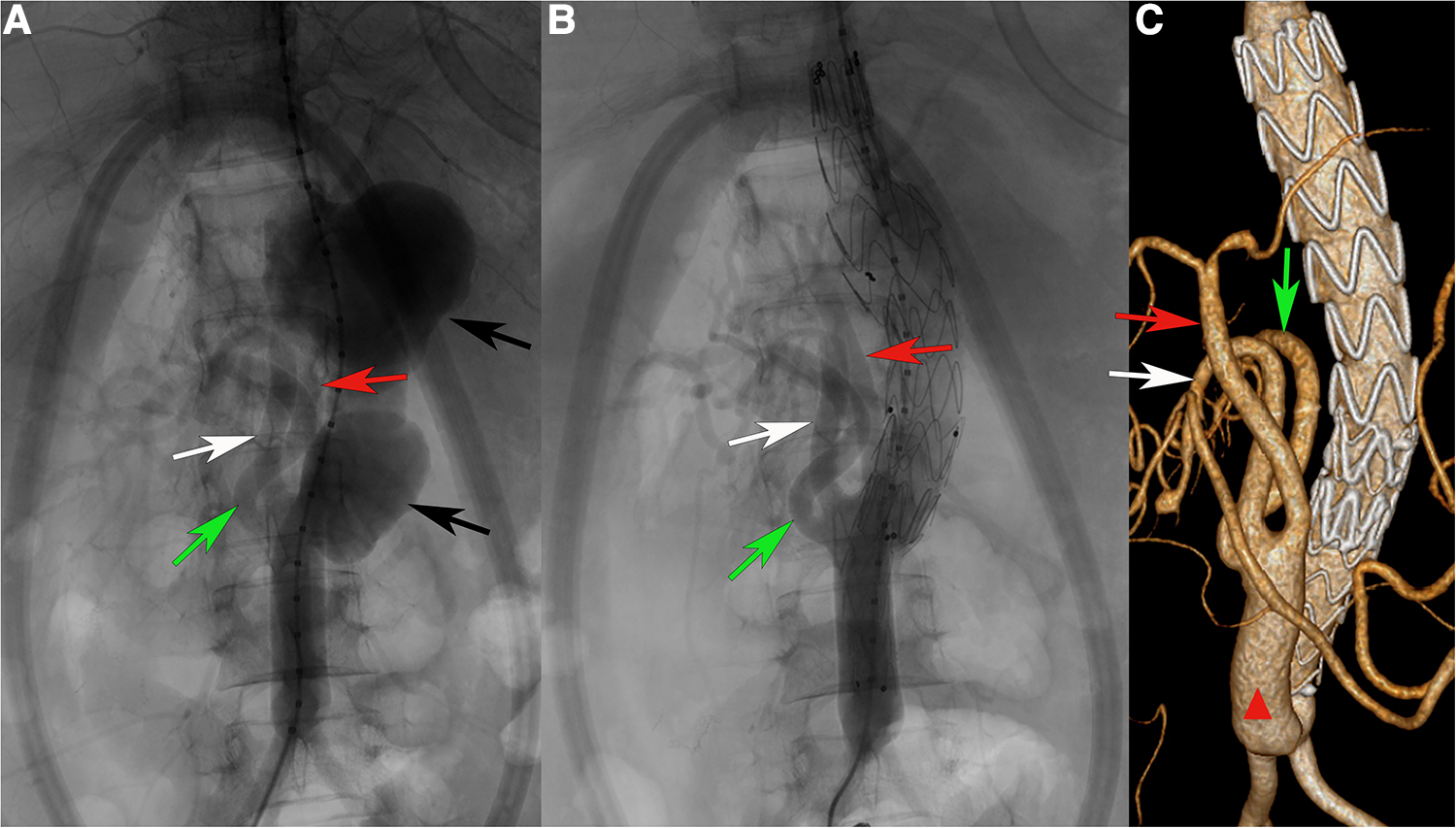
(**A**) Angiography after completion of splanchnic artery branch reconstruction. (**B**) Angiography after completion of stent placement. (**C**) After 5 years of follow-up, CTA showed that the pseudoaneurysm cavity completely disappeared, and the stent and branch arteries were patent. Green arrow, right renal artery. White arrow, superior mesenteric artery. Red arrow, celiac artery. Black arrow, abdominal aortic pseudoaneurysms. Red triangle: Artificial blood vessel.

A 32-year-old female presented with a chief complaint of tearing pain in the back for 2 weeks with persistent worsening. The patient was thin with a malnourished appearance. Physical examination indicated a postoperative scar of the abdomen. Biochemical indexes indicated that serum albumin was 36 g/L, hemoglobin 102 g/L, erythrocyte sedimentation rate (ESR) 35 mm/h, and procalcitonin 0.3 ng/ml had no other abnormalities. Computerized topographic angiography (CTA) reported that a thoracoabdominal aortic aneurysm and an abdominal aortic aneurysm were located in the proximal and distal artificial vessel (5.5 cm and 3.0 cm, respectively, Figure 1A, left one), respectively. In addition, she had no smoking or drinking history and no dental or genital ulcer. However, the patient had a very complex and repeated history of aneurysms and surgery.

**Figure 2:**
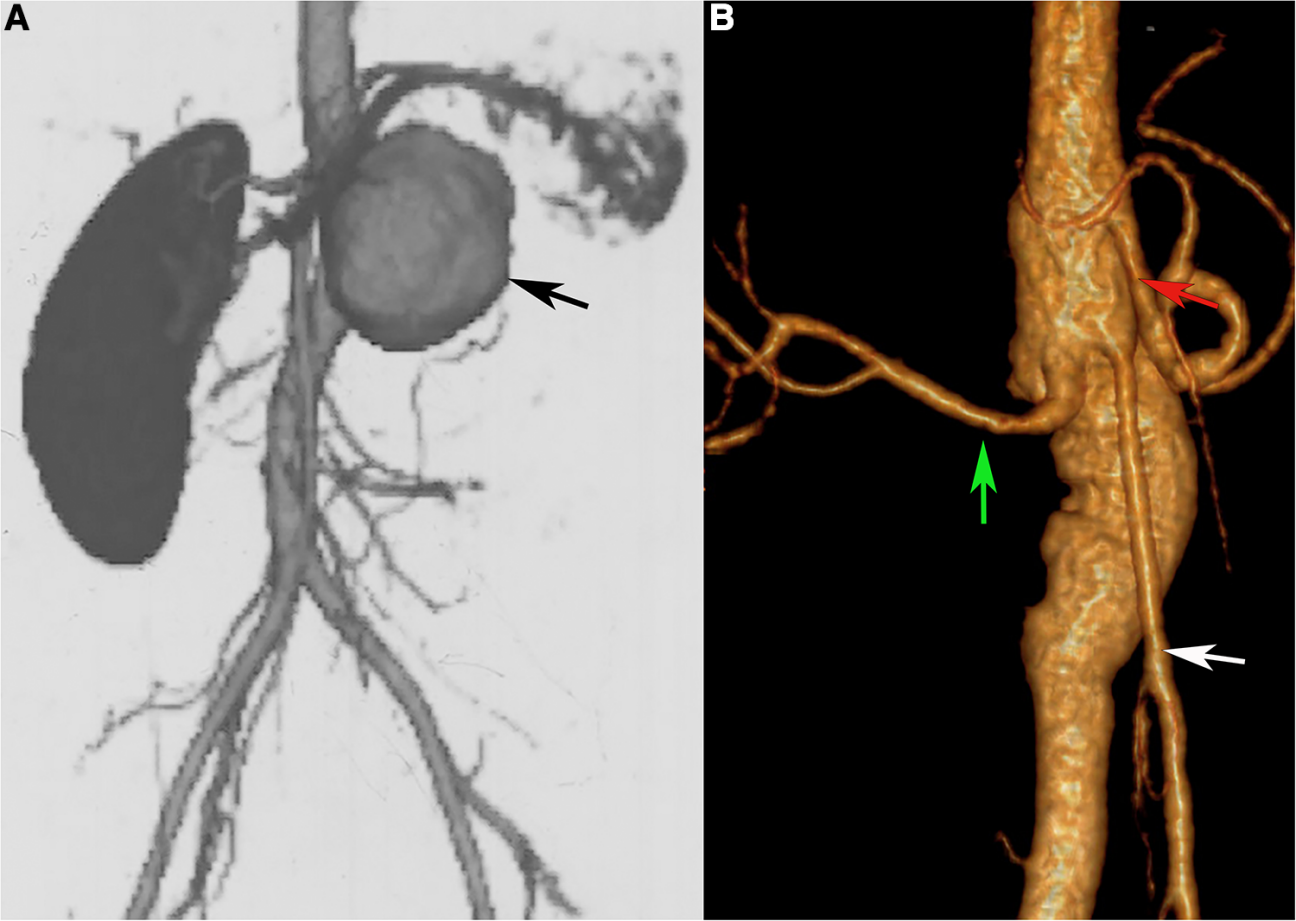
(**A**) abdominal aortic pseudoaneurysm (black arrow). (**B**) CT Angiography after pseudoaneurysm resection and abdominal aorta reconstruction. Green arrow, right renal artery. White arrow, superior mesenteric artery. Red arrow, celiac artery. Black arrow, abdominal aortic pseudoaneurysms.

She had undergone left nephrectomy and renal artery ligation approximately 19 years ago because of a giant left renal aneurysm with a diameter of 10 cm at a local hospital. Unfortunately, 13 years ago, she underwent another open repair for a juxtarenal abdominal aortic aneurysm (6.0*5.3 cm in diameter, Figure 2A. Figure 2B-postoperative CTA). She was diagnosed with Vasculo-Behcet's disease after the second aneurysm procedure and accepted immunosuppressive therapy. However, 7 months after this operation, the patient experienced epigastric pain and vomiting after eating, which were relieved by symptomatic treatment. Ultimately, the patient developed diffuse peritonitis, and open exploration confirmed small bowel necrosis due to intestinal volvulus, and resection of the necrotic small bowel was performed. It was thought to be caused by multiple abdominal operations resulting in adherent ileus and then leading to strangulated ileus. Despite multiple episodes of aneurysm, immunological tests did not reveal abnormalities.

To minimize the occurrence of postoperative complications, preoperative immunosuppressive therapy and nutritional support were intensified. During this period, the patient's vital signs and symptoms were closely monitored in preparation for emergency surgical treatment at any time. For this recurrent aneurysm, completely open repair was challenging due to the history of multiple surgeries. Facing the celiac trunk, superior mesenteric artery, and right renal artery, fenestrated endovascular repair or chimney stents are difficult and very costly in terms of medical care. Due to thoracoabdominal aortic aneurysm, reconstruction is time consuming, and the organ has a high risk of ischemic damage and even necrosis in traditional open surgery. The patient was young and had undergone multiple surgeries. To shorten the duration of aortic cross-clamping and end-organ ischemia, hybrid surgery was performed (artificial vessel shaping *in vitro*-bypass-endovascular stent repair). When the internal environment improved, we started the surgical treatment. Prior reconstruction used an artificial vessel stent to reconstruct the abdominal trunk, the right renal artery, and the superior mesenteric artery (16 × 8 mm bifurcated artificial vessel was reshaped into three bifurcations), anastomosed the artificial vessel to the aorta at the normal vessel underneath the second abdominal aortic aneurysm, and then reconstructed the branch arteries sequentially, which greatly reduced the organ ischemia time. After reconstruction was completed (Figure 1A, left), the first stent (Medtronic ENEW2020C80) was released above the artificial vessel anastomosis. The second stent (VAMC2622C150TE) was released in the middle of the first stent, with the proximal end of the stent located to the normal aorta located superior to the thoracoabdominal aortic aneurysm (Figure 1A, right).

The patient was discharged on antiplatelet therapy (100 mg aspirin and 40 mg glucocorticoid) and regular immune therapy in the rheumatology ward. During her 60-month follow-up, the graft had no displacement or internal leakage, and the reconstructed blood vessels were unobstructed (Figure 1C).

## Discussion

Behcet's disease (BD) is considered a syndrome rather than a disease and is a systemic variable vessel vasculitis that involves many systems (8, 15). It has the highest prevalence along the ancient silk road, which stretches from the Mediterranean through the Middle East to East Asia (16). BD usually starts during the second or third decade of life, and an early age of disease onset seems to be a poor prognostic factor in addition to male sex (17). The majority of patients (75%) experience their first vascular event within 5 years of disease onset (6). Vascular disease develops in up to 25% to 35% of patients and has a definite male preponderance (6, 18).

Immunosuppressive therapy, such as cyclophosphamide, corticosteroids, and even monoclonal anti-TNF antibodies, is the main medical treatment for BD aneurysms (8, 19). Medical therapy should be started as early as possible to provide a stable internal environment for surgical treatment and reduce the incidence of postoperative complications (20). The timing of surgery depends on the size of the aneurysm and the patient's symptoms.

At present, open surgery or endovascular repair is still the main surgical method for aneurysms or pseudoaneurysms. Medical treatment combined with endovascular aneurysm or pseudoaneurysm exclusion in BD is feasible and effective (21–23). The graft occlusion rate despite immunosuppressive treatment was approximately 40% in patients who received bypass surgery (18). In addition, the incidence of pseudoaneurysm formation at the anastomosis, stent anchoring area or access site for stent graft introduction and graft occlusion is high. Several patients accepted intervention again because pseudoaneurysms or aneurysms occurred at other sites (24, 25). Previous studies indicated that most patients died from aneurysm or pseudoaneurysm rupture after surgery (21, 25, 26). Therefore, reducing the occurrence of postoperative aneurysms and pseudoaneurysms is the key to improving the prognosis. Mousa et al. proposed that mechanical prosthetic wrapping for vascular anastomoses could reduce postoperative pseudoaneurysm formation after aortic aneurysm in BD, and his study showed a 6.3% (1/16) incidence of postoperative anastomotic pseudoaneurysm. The surgeries in BS patients had a large risk of complications such as wound dehiscence, infection, or graft failure. Implementing immunosuppressive treatment can reduce the occurrence of postoperative complications. The results of the pathology test can help to guide the initiation of postoperative immunotherapy (27). Hong et al. reported a successful case-hybrid endovascular repair of thoracic aortic aneurysm in a patient with Behcet's disease following right to left carotid-carotid bypass grafting in 2011 (28).

In our case, the patient underwent three procedures for aneurysms: the first was resection of the large renal aneurysm and left kidney, the second was open surgery on the proximal renal abdominal aortic aneurysm, and the third was hybrid surgery for the pseudoaneurysm of the proximal and distal anastomosis. We speculated that the postoperative pseudoaneurysm that formed after the second procedure was due to the inadequate length of the proximal and distal resected abdominal aorta and the active inflammatory period, which resulted in the abnormal vessel still existing at the anastomosis. To avoid the formation of another pseudoaneurysm at the anastomosis, adequate preparation was performed before surgery. The hybridization procedure, retrograde visceral artery bypass and endovascular repair, was performed. This surgical approach allowed for less clamping of the suprarenal abdominal aorta and could better maintain intraoperative circulatory stability. After reconstruction of the major visceral artery, the length of the stent anchorage zone could be increased. However, it requires a normal abdominal aorta distal to the aneurysm, and retrograde bypass can cause changes in hemodynamic forces that may cause a slowing of blood flow. In addition, the location of the arterial bypass anastomosis is at risk of reappearing as a pseudoaneurysm and consequently a fatal attack.

According to current reports (29, 30), fenestrated-branched endovascular aortic repair presents another option for treating complex abdominal aortic aneurysms. Despite the high technical success rate and low perioperative mortality, unsatisfactory medium- and long-term prognosis as well as high re-intervention rates have been observed (30). The overall cost of total endovascular surgery without further intervention is already very expensive (31). In view of the above situation, we choose the hybrid operation program. And the excellent follow-up results of the patient's five years after surgery-no recurrence of aneurysm or pseudoaneurysm, no graft infection, and no target vessel occlusion suggested that a hybrid surgical approach can also become a treatment option for Behcet's disease-associated aneurysms.

## Conclusion

Hybrid surgery may be a feasible and effective strategy for BD aneurysms. Adequate preoperative and postoperative immunotherapy with arterial anastomosis away from the diseased artery may be the key to success.

## Data Availability

The original contributions presented in the study are included in the article/Supplementary Material, further inquiries can be directed to the corresponding author.
